# Supplementary material for: Potential Distribution of Invasive Boxwood Blight Pathogen (Calonectria pseudonaviculata) as Predicted by Process-Based and Correlative Models
Source: Biology (Basel). 2022 Jun 1;11(6):849. doi: 10.3390/biology11060849 (PMC9220671; doi:10.3390/biology11060849)
Supplement: Supplementary file 1 [file biology-11-00849-s001.zip › Supporting_Info_BOXB_SDM2.pdf]

Supplementary Information

Original research manuscript submmited to Biology

Title: Potential distribution of invasive boxwood blight pathogen (*Calonectria pseudonaviculata*) as predicted by process-based and correlative models.

Authors: Brittany S. Barker [1,2,*], Leonard Coop [1,2] and Chuanxue Hong [3]

[1] Oregon Integrated Pest Management Center, 4575 Research Way, Oregon State University, Corvallis, OR 97331, USA; brittany.barker@oregonstate.edu (B.B.); coopl@oregonstate.edu (L.C.)
[2] Department of Horticulture, Oregon State University, 4017 Agriculture and Life Sciences Building, Oregon State University, Corvallis, OR 97331, USA
[3] Hampton Roads Agricultural Research and Extension Center, Virginia Polytechnic Institute and State University, 1444 Diamond Springs Road, Virginia Beach, VA 23455, USA; chhong2@vt.edu (C.H.)
* Correspondence: brittany.barker@oregonstate.edu (B.B.)

Figure S1. Known global distribution of *Calonectria pseudonaviculata* according to EPPO [1]. Map was last updated on 2020-11-24.

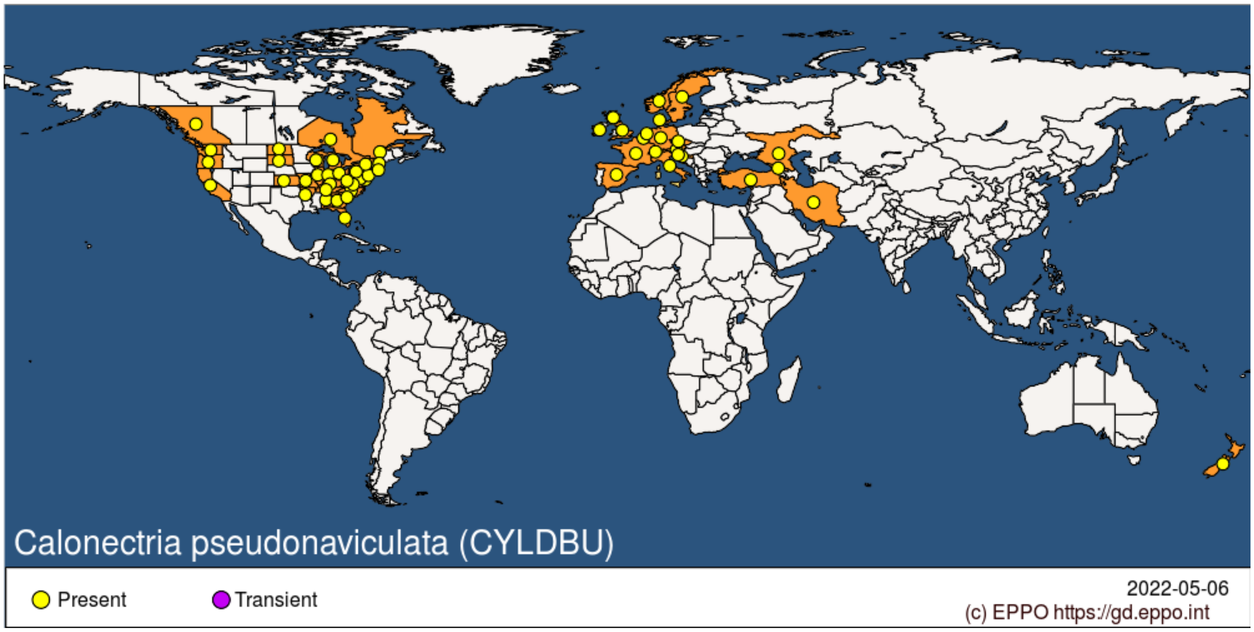

Figure S2. Eigenvectors (loadings) of the principal component analysis (PCA) of 27 bioclimatic variables used for producing correlative models. The direction and length of the arrows illustrate the contribution of the variables to the first two axes of the PCA. Abbreviations: b1: annual mean temperature; b2: mean diurnal temperature range; b3: isothermality; b4: temperature seasonality; b5: max temperature of warmest week; b6: min temperature of coldest week; b7: temperature annual range; b8: mean temperature of wettest quarter; b9: mean temperature of driest quarter; b10: mean temperature of warmest quarter; b11: mean temperature of coldest quarter; b12: annual precipitation; b13: precipitation of wettest week; b14: precipitation of driest week; b15: precipitation seasonality; b16: precipitation of wettest quarter; b17: precipitation of driest quarter; b18: precipitation of warmest quarter; b19: precipitation of coldest quarter; b28: annual mean moisture index; b29: highest weekly moisture index; b30: lowest weekly moisture index; b31: moisture index seasonality; b32: mean moisture index of wettest quarter; b33: mean moisture index of driest quarter; b34: mean moisture index of warmest quarter; b35: mean moisture index of coldest quarter.

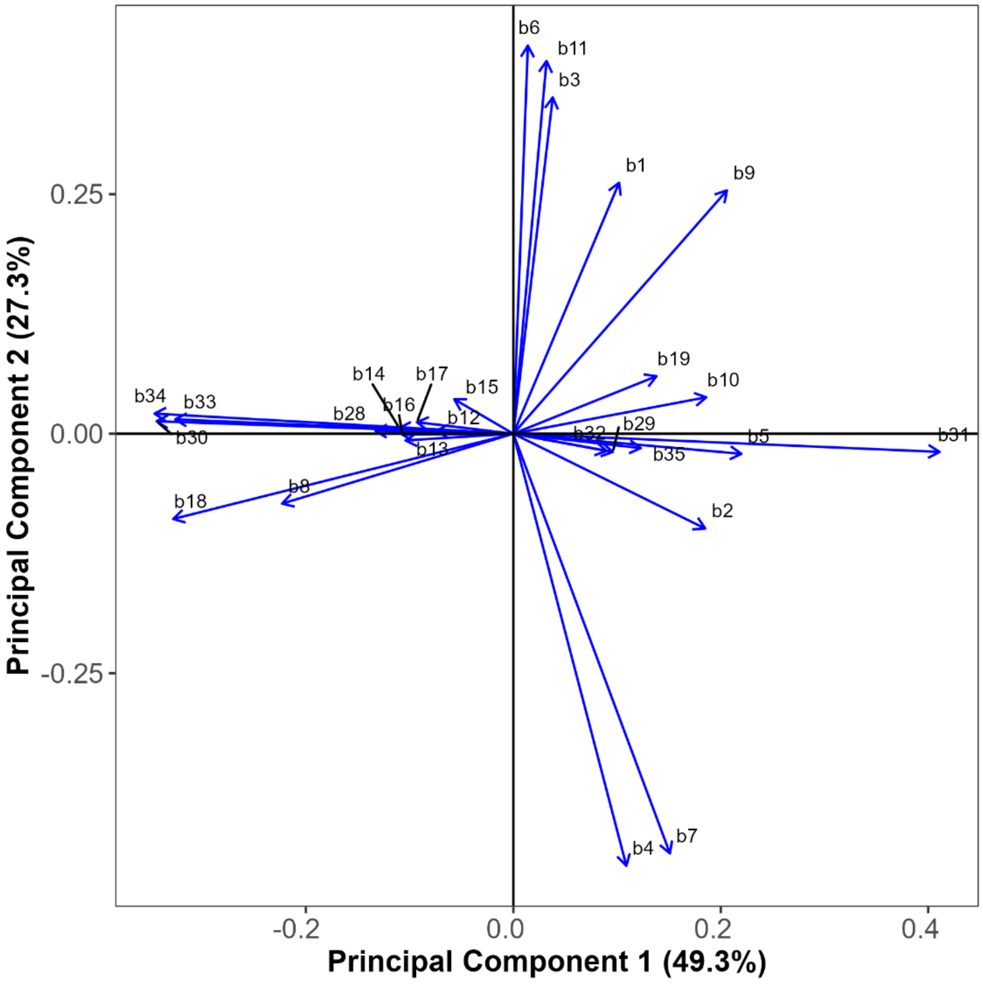

Figure S3. Mobility-oriented parity (MOP) assessment outputs for correlative model projections. Areas with MOP metric values close to 1 (warm colors) have highly comparable climatic conditions to those in the model calibration area. Areas with values approaching 0 (cool colors) indicate higher extrapolation because one or more climatic variables have values outside the range of variable(s) in the calibration area.

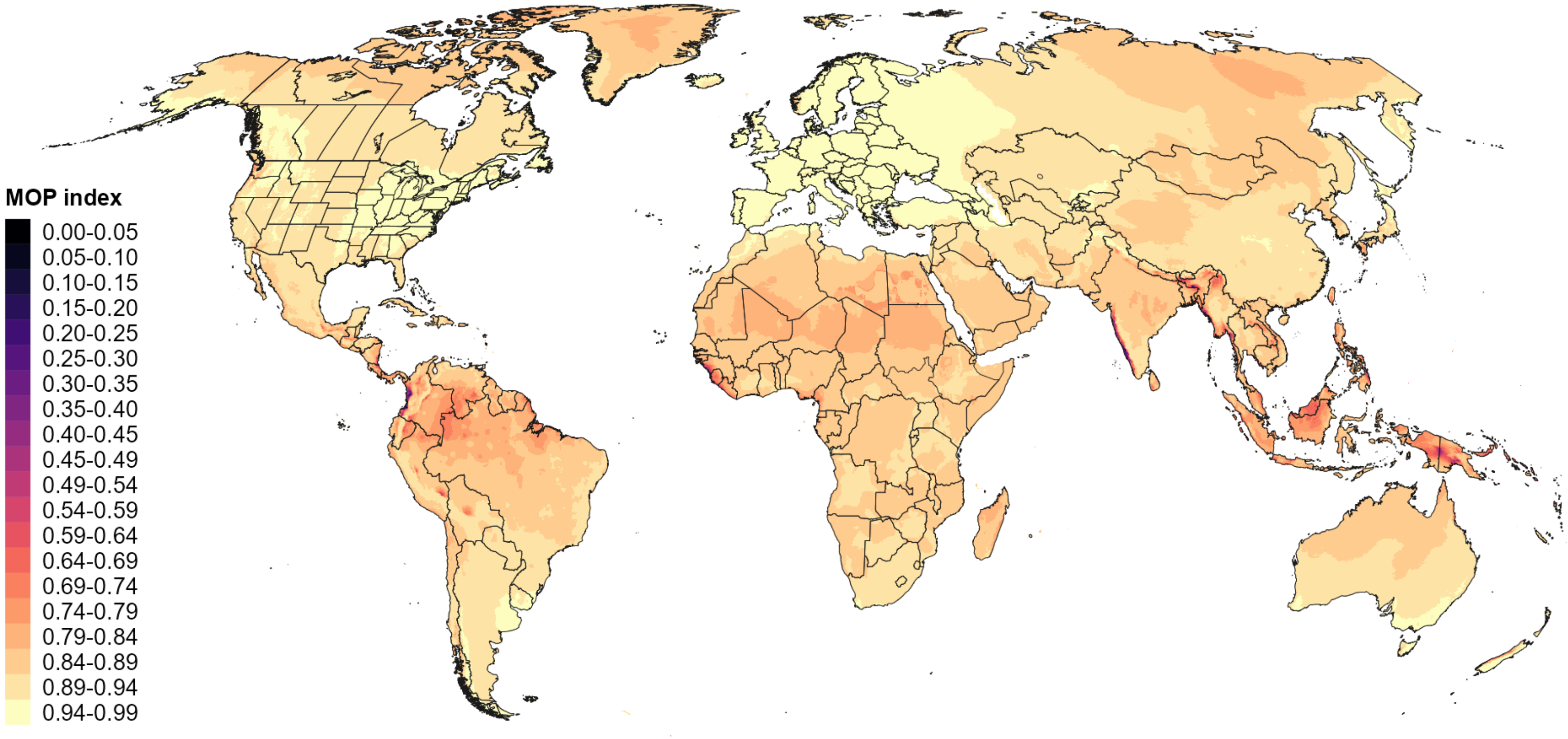

Figure S4. Maps of climatic suitability for *Calonectria pseudonaviculata* in New Zealand. Climatic suitability is estimated as the ecoclimatic index in the (a) CLIMEX model and as the probability of occurrence in (b–f) correlative models produced using boosted regression trees (BRT), Gaussian process (GAU), Maxent "simple" (MXS), random forest (RDF), and a principal component analysis of predictions (ensemble) produced by the four algorithms (ENS-PCA). Black circles in the CLIMEX map depict the approximate location of presence records.

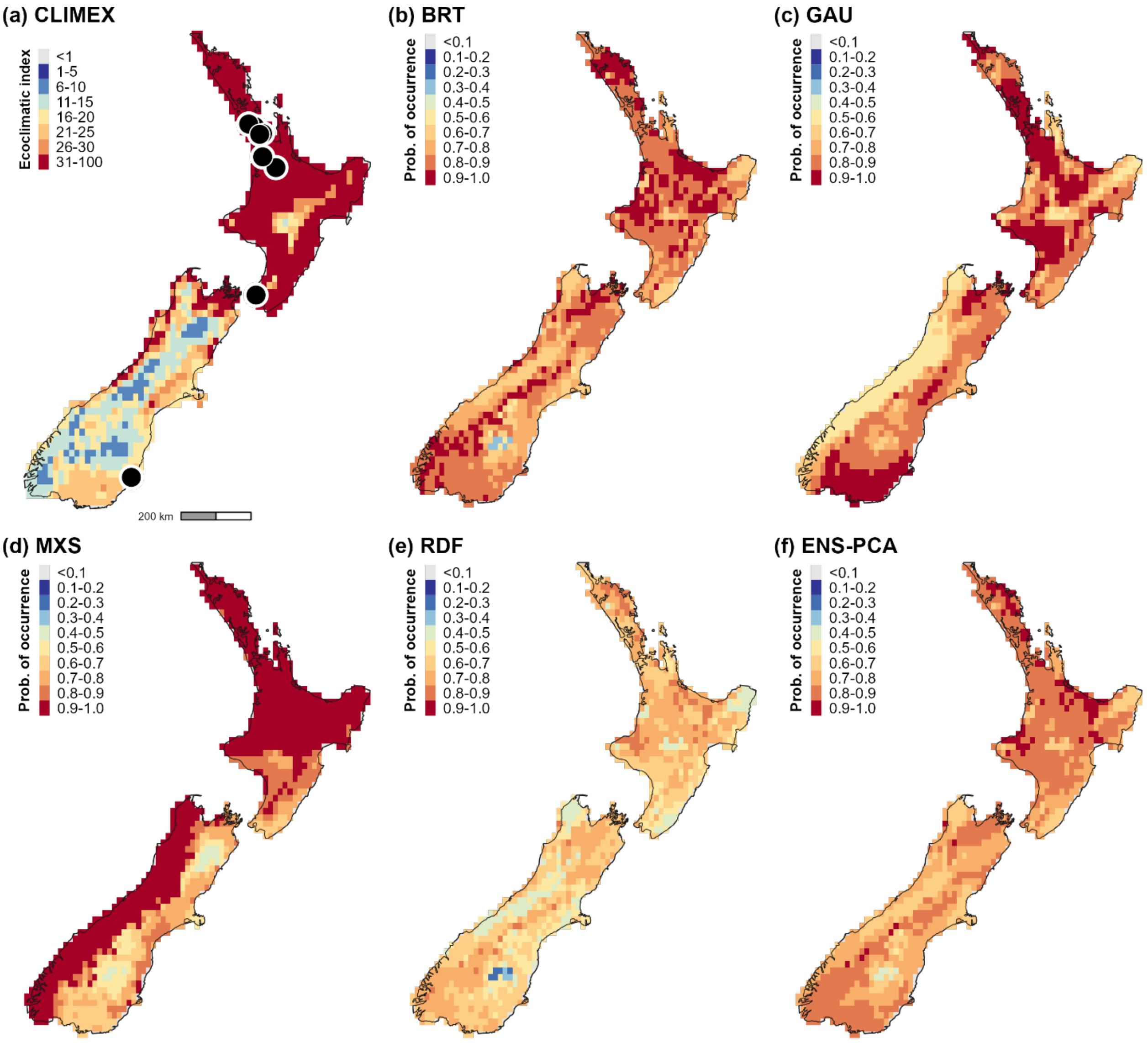

Figure S5. Maps depicting predictions of the accumulation of (**a**) cold stress, (**b**) heat stress, and (**c**) dry stress at a global scale produced by CLIMEX.

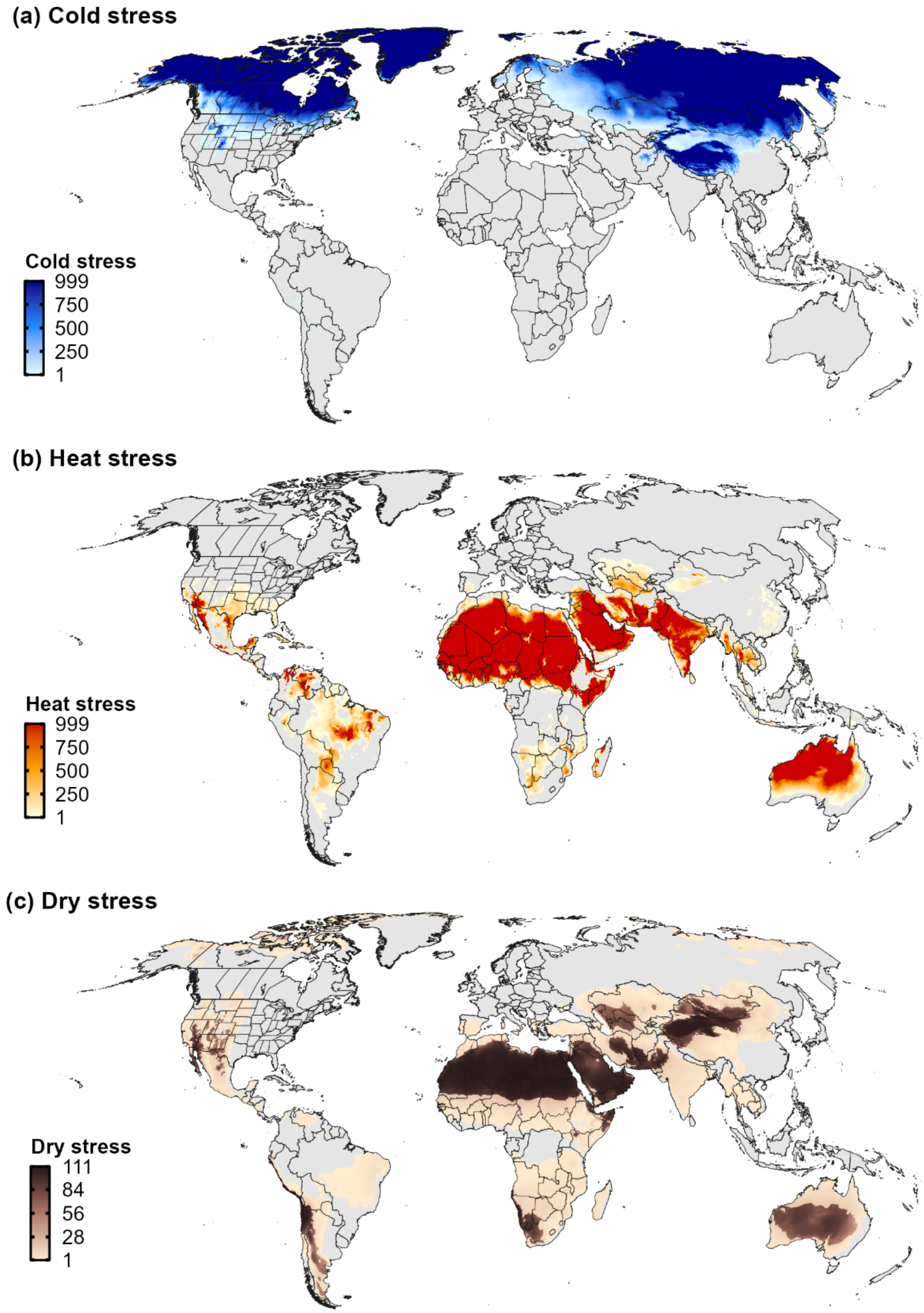

References

1. EPPO. *Calonectria pseudonaviculata* (CYLDBU) Available online: https://gd.eppo.int/taxon/CYLDBU/distribution (accessed on 5 May 2022).
